# Orthodontic bonding to silicate ceramics: impact of different pretreatment methods on shear bond strength between ceramic restorations and ceramic brackets

**DOI:** 10.1007/s00784-021-04260-5

**Published:** 2021-11-18

**Authors:** Rebecca Jungbauer, Christian Kirschneck, Christian M. Hammer, Peter Proff, Daniel Edelhoff, Bogna Stawarczyk

**Affiliations:** 1 Department of Orthodontics, University Medical Centre Regensburg, Regensburg, Germany; 2 Department of Prosthetic Dentistry, University Hospital, LMU Munich, Germany; 3 Institute of Functional and Clinical Anatomy, Friedrich Alexander University Erlangen-Nürnberg, Erlangen, Germany

**Keywords:** Silicate ceramic, Orthodontic bonding, Shear bond strength, Ceramic bracket, Pretreatment

## Abstract

**Objective:**

The study aims to investigate the shear bond strength (SBS) between silicate ceramic restorations and ceramic brackets after different pretreatments and aging methods.

**Material and methods:**

Leucite (LEU) and lithium disilicate (LiSi) specimens were pretreated with (i) 4% hydrofluoric acid + silane (HF), (ii) Monobond Etch&Prime (MEP), (iii) silicatization + silane (CoJet), and (iv) SiC grinder + silane (SiC). Molars etched (phosphoric acid) and conditioned acted as comparison group. SBS was measured after 24 h (distilled water, 37 °C), 500 × thermocycling (5/55 °C), and 90 days (distilled water, 37 °C). Data was analyzed using Shapiro–Wilk, Kruskal–Wallis with Dunn’s post hoc test and Bonferroni correction, Mann–Whitney *U*, and Chi^2^ test (*p* < 0.05). The adhesive remnant index (ARI) was determined.

**Results:**

LEU pretreated with MEP showed lower SBS than pretreated with HF, CoJet, or SiC. LiSi pretreated with MEP resulted in lower initial SBS than pretreated with HF or SiC. After thermocycling, pretreatment using MEP led to lower SBS than with CoJet. Within LiSi group, after 90 days, the pretreatment using SiC resulted in lowest SBS values. After HF and MEP pretreatment, LEU showed lower initial SBS than LiSi. After 90 days of water storage, within specimens pretreated using CoJet or SiC showed LEU higher SBS than LiSi. Enamel presented higher or comparable SBS values to LEU and LiSi. With exception of MEP pretreatment, ARI 3 was predominantly observed, regardless the substrate, pretreatment, and aging level.

**Conclusions:**

MEP pretreatment presented the lowest SBS values, regardless the silicate ceramic and aging level. Further research is necessary.

**Clinical relevance:**

There is no need for intraoral application of HF for orthodontic treatment.

## Introduction

The number of adult patients asking for orthodontic treatment is steadily increasing [1, 2]. In many cases treatment with fixed appliances is necessary to accomplish a fully controlled movement of the teeth in all three dimensions. Therefore, brackets need to be bonded temporarily to the enamel surface after a combination of etching using 35% phosphoric acid and conditioning using an adhesive system. The recommended bond strength in the literature is 5–10 MPa [3]. In those cases, where patients present with existing dental restorations, the orthodontist needs a reliable treatment protocol for bonding brackets to artificial surfaces, in many cases tooth colored restorations such as reinforced (lithium silicate, LiSi) and low reinforced silicate (leucite, LEU) ceramics. Both type of ceramics are silicate ceramics, which have a very high translucency compared to zirconia ceramic and are therefore very esthetic [4, 5]. Lithium silicate ceramics show higher flexural strength and fracture toughness compared to leucite-based ceramics, based on the higher volume fraction of crystals and therefore a tighter interlocking matrix of the silicate-based materials [6–8]. An interaction between the bonding material and the microporosity of the ceramic is determined by the capability of the resin cement to wet the ceramic surface, dependent on the surface chemistry and roughness of the ceramic as well as the viscosity and composition of the bonding material [9–11]. The surface roughness of the ceramic is affected by etching or mechanical treatment. During orthodontic treatment the number of accidental bracket losses must be kept to a minimum, but at the same time the silicate ceramic surface should not be damaged when brackets are removed after active treatment [12, 13]. In the literature the intraoral application of 5–9% hydrofluoric acid (HF) for orthodontic purposes for 60 s in combination with the use of a silane is often described to prepare silicate surfaces and create micromechanical and chemical retention for bonding brackets [14–22]. The general recommendation is to etch only low reinforced silicate ceramics for 60 s with HF. In contrast, reinforced ceramics such as lithium silicate ceramics should only be etched for 20–30 s [23]. This reinforced ceramic contains less glass phase for the HF etching process. Therefore, with longer etching treatment times, the surface becomes smoother again, resulting in reduced adhesion. Due to the different etching times of silicate ceramic types, the practitioner needs to know which type of ceramic is present to attach the brackets to. Apart from this procedure being very time consuming [24], HF is known to be very toxic and can cause various hazardous effects for both the patient and even more the clinician who deals with HF more frequently [25–28]. Nevertheless, the application of HF seems to be the actual gold standard to prepare silicate ceramic surfaces to bond brackets [22, 29]. From the clinician’s point of view an all-in-one pretreatment with a reduced hazard potential for patients and clinicians, such as Monobond Etch&Prime (MEP), would be desirable for bonding brackets to silicate ceramic restorations. Furthermore, different pretreatment methods, which are used in restorative and prosthetic dentistry for intraoral reparation of silicate ceramic restorations without the application of any acids, are viable options for orthodontic purposes. These comprise mechanical roughening of the surface with silicium carbide (SiC) grinders or chemical/mechanical pretreatment with intraoral silicate coating (CoJet) [30–32]. According to the DIN 13,990:2017–04 testing the SBS values after 500 thermal cycles is recommended additional to the initial ones [33]. Therefore, the aim of this study was to investigate the shear bond strength of ceramic brackets and two types of silicate ceramic surfaces after different pretreatment methods. The assumed hypotheses were that (1) the pretreatment of the silicate ceramic, (2) the aging level, and (3) the choice of silicate ceramic show no impact on shear bond strength values.

## Materials and methods

### Specimen preparation

One-hundred-eighty square specimens were cut into slices of 3-mm thickness with a low-speed diamond saw under constant water application (Secotom-50, Struers, Ballerup, Denmark) from lithium disilicate blanks (LiSi, IPS e.max CAD A2/C14, Ivoclar Vivadent, Schaan, Liechtenstein). LiSi specimens in the purple state underwent a specific treatment for final crystallization (program IPS e.max CAD Crystal/Glaze HT/LT, furnace: Programat EP 5000, Ivoclar Vivadent; Table 1). The 180 leucite ceramic specimens (LEU, VITA VM 13, VITA, Bad Säckingen, Germany) were fabricated using layering technique. To achieve standardized size of the specimens, a silicone key was used. In a second firing, under the same conditions, dentin was added to compensate for the shrinkage of the sintering process. Prior to the second firing (Austromat 654, preee-i-dent, Dekema, Freilassing, Germany; Table 1), the slurry was condensed into the mold using a vibrator for 2 s at 50 Hz (ElektroVibrator Porex, Renfert, Hilzingen, Germany).

**Table 1:** Firing schedules of the ceramics

Veneering ceramic	Pre drying		Heating rate (°C/min)	Firing temperature (°C)	Holding time (min)	Vacuum during heating
	Temperature (°C)	Time (min)				
VITA VM13	500	6	55	890	1	Yes
IPS e-max CAD	403	6	60	850	10	Yes

Thereafter, all 360 specimens were embedded in acrylic resin (ScandiQuick A and B; Scan-Dia, Hagen, Germany) and polished up to P2000 (SiC paper; Struers) with an automatic polishing machine (Tegramin 20, Struers) under continuous water-cooling. The specimens were randomly assigned to one of the 12 subgroups, resulting in 24 groups (15 specimens in each group in accordance with previous studies [34–37]). As comparison and to be able to quantify the SBS value, 45 human third molars free of caries and restorations were collected after extraction, stored in 0.5% chloramine-T solution (Sigma-Aldrich Laborchemikalien, Seelze, Germany; Lot No. 53110) for 1 week at room temperature, and stored in distilled water at 5 °C for a maximum of 6 months. They were fixed with the buccal side up and parallel to the base with dental technician wax and embedded in the acrylic resin (ScandiQuick A and B). The collection and use of human teeth extracted for medical reasons was approved by the ethics committee of the University of Regensburg, Germany (12–170-0150). All methods were carried out according to relevant regulations and guidelines. From all participants or their parent/legal guardian informed consent was obtained.

### Ceramic pretreatment and bracket bonding

**Fig. 1:**
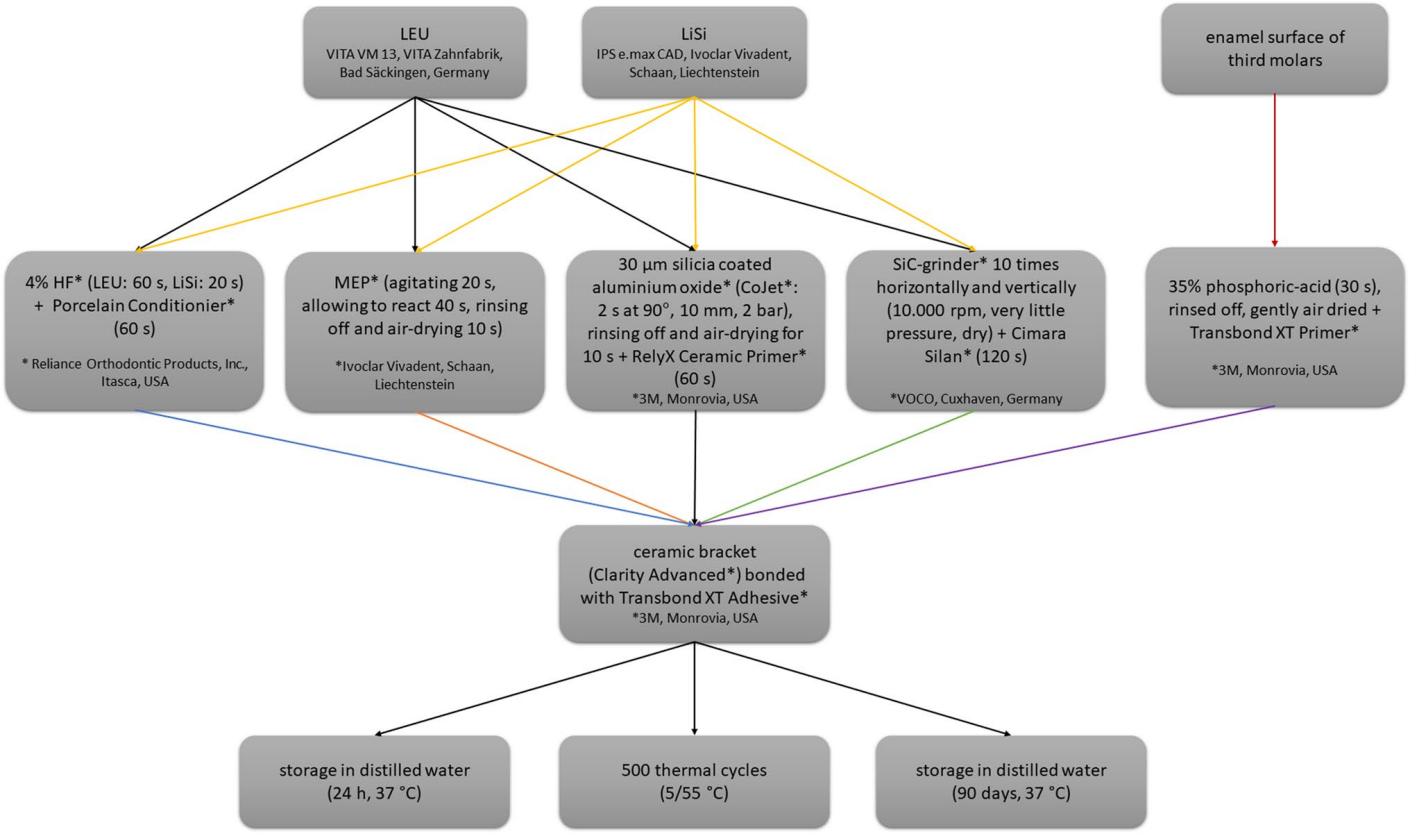
Study workflow. The flowchart presents the pretreatment of the two different ceramic and enamel specimens. *LEU* leucite, *LiSi* lithium disilicate, *HF* hydrofluoric acid, *MEP* Monobond Etch&Prime, *SiC grinder* silicium carbide grinder

Before pretreatment, all specimens were scrubbed with a pumice/water mixture (40 g:50 g) and a polishing brush (Busch & Co., Engelskirchen, Germany) for 3 s moving from left to right and 3 s up and down at a speed of 3000 rounds per minute. Afterward the pumice was rinsed off with water. Depending on the group the ceramic surface was pretreated as shown in Fig. 1. The third molars were conditioned with 35% phosphoric acid (iBond Etch, Kulzer, Hanau) for 30 s before rinsing off the acid with water and gently air drying until a frosty surface was visible. A thin layer of Transbond XT Primer (3 M, Monrovia, USA) was applied to the surface and gently dispersed with compressed air. The ceramic brackets (Clarity Advanced, 3 M, Monrovia, USA) were bonded directly to each specimen.

After placing the bracket, gentle pressure was applied to keep the interface of the attachment material to a minimum. Excess was removed carefully with a dental probe before 3 s of light-curing (1600 mW/cm^2^, Ortholux luminous curing light, 3 M, Monrovia, USA) through the center of the ceramic bracket. After the bonding procedure, all specimens were directly stored in distilled water. Shear bond strength was tested either after 24 h wet storage in distilled water at 37 °C (baseline/initial), 500 thermal cycles (5/55 °C, dwelling time: 20 s, Thermocycler THE 1100, SD Mechatronik, Feldkirchen-Westerham, Germany), or 90 days wet storage in distilled water at 37 °C. Detailed information on materials used is given in Table 2.

**Table 2:** Manufacturer and composition of the luting materials employed in this study

Materials	Manufacturer	Composition
PorcEtch (HF)	Reliance Orthodontic Products	Hydrofluoric acid (7%), aqueous solutions, sodium fluoride
Porcelain conditioner	Reliance Orthodontic Products	Ethanol/denatured, 3-(trimethoxysilyl)propyl-2-methyl-2-propenoic acid, acetic acid
Monobond Etch&Prime (MEP)	Ivoclar Vivadent	Tetrabutyl ammonium dihydrogen trifluoride, methacrylated phosphoric-acid ester, trimethoxysilylpropyl methacrylate, alcohol, water
RelyX Ceramic Primer	3 M	Ethyl alcohol, water, methacryloxypropyltrimethoxysilane
Cimara Silan	VOCO	Mixture of various dimethacrylates, initiators, 2-propanol, silicates, additives
TransbondXT primer	3 M	Bis-GMA, TEGDMA, triphenylantimony, 4-(dimethylamino)-benzeneethanol, DL-camphorquinone, hydroquinone
TransbondXT adhesive	3 M	Silane-treated quartz, Bis-GMA, EBPADMA, silane-treated silica, diphnyliodonium hexafluorophosphate

*Bis-GMA* bisphenol-A-diglycidyl ether dimethacrylate, *TEGDMA* triethylene glycol dimethacrylate, *EBPADMA* bisphenol-A-bis(2-hydroxyethyl ether) dimethacrylate

### Shear bond strength testing

**Fig. 2:**
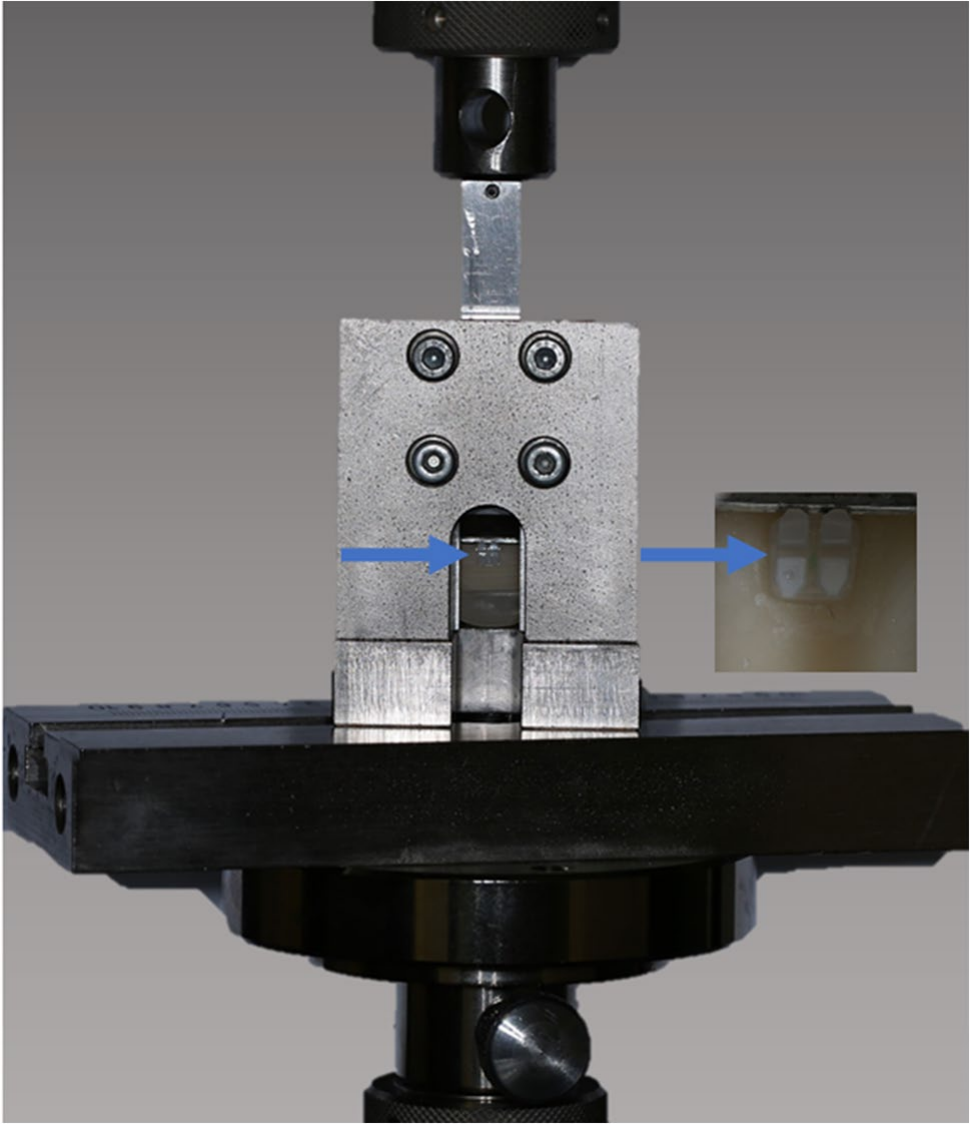
Experimental setup for SBS measurements. The specimen with the bonded bracket (arrow and in higher magnification) is fixed in the test apparatus and placed in the universal testing machine. The force is applied in an occlusal-gingival direction (downward) and recorded simultaneously

Before testing all specimens were stored in distilled water at room temperature for 1 h and shear bond strength was tested at room temperature (23 °C). The gently dried specimens were placed in a specially designed test apparatus (Fig. 2) in the universal testing machine (RetroLine, Zwick/Roell, Ulm, Germany). The compressive force was applied perpendicularly to the specimen surface in an occluso-gingival direction with a crosshead speed of 1 mm/min until fracture. The maximum force was recorded in Newton and the area of the bracket was given by the manufacturer. SBS was calculated using the formula:

$$\mathrm{R }(\mathrm{N}/\mathrm{mm}2)=\frac{F (N)}{A ({\mathrm{mm}}^{2})}$$

After the SBS testing, all specimens were photographed using a microscope with 10 × magnification (Bresser, Rhede, Germany) to search for cracks and to determine the adhesive remnant index (ARI). ARI was evaluated as follows: 0 = no remaining attachment material (AM) on the ceramic, 1 = less than 50% remaining on the ceramic, 2 = more than 50% remaining on the ceramic, and 3 = 100% AM remaining on the ceramic [38]. Additionally, seven specimens were viewed with a JEOL scanning electron microscope at 30 × and 250 × magnification (JSM-IT 300LV, JEOL Germany, Eching, Germany) after sputter-coating with a 20-nm layer of gold using the Leica EM ACE200 system (Leica Mikrosysteme, Vienna, Austria).

### Reliability of measurements

To assess intrarater and interrater reliability of the ARI the Cohen’s kappa coefficient (*κ*) was calculated. Therefore, 20 specimens were selected to determine the ARI a second time by the same investigator and additionally by a second experienced investigator.

### Statistical methods

The software IBM SPSS Statistics 23 (IBM, Armonk, NY, USA) was used for statistical analysis. For the descriptive statistic medians (MD), interquartile ranges (IQR) as well as minimum (MIN) and maximum (MAX) were reported. According to a Shapiro–Wilk test and visual assessment of the histograms more than 5% of the data were not distributed normally. Therefore, non-parametric Kruskal–Wallis tests by ranks, followed by pairwise comparison Dunn’s post hoc tests with *p*-value Bonferroni correction and Mann–Whitney *U* tests were applied to check the differences between groups for statistical significance. The Chi^2^ test was used to evaluate the ARI. *P* values ≤ 0.05 were considered as statistically significant.

## Results

### Influence of pretreatment

Within LEU ceramic, MEP pretreatment (23.2 MPa) presented lower initial SBS than CoJet (37.0 MPa, *p* = 0.001) or SiC (40.9 MPa, *p* < 0.001). After aging of 500 thermal cycles, MEP pretreatment (17.7 MPa) resulted in lower SBS values compared to HF (33.7 MPa, *p* = 0.005), CoJet (40.6 MPa, *p* < 0.001), and SiC (35.3 MPa, *p* < 0.001; Table 3).

**Table 3:** Non-parametric descriptive statistics including median, interquartile range (IQR), minimum (Min), and maximum (Max). All values are in MPa

Pretreatment	Aging	LEU				LiSi			
		Median	IQR	Min	Max	Median	IQR	Min	Max
HF	24 h	33.6 ^ab, A, 1^	11.7	18.5	53.4	40.4 ^a, B, 1^	17.1	28.8	59.4
MEP		23.2 ^a, C, 2, 3^	7.5	10.2	41.4	35.7 ^b, D, 2^	13,7	12.7	48.5
CoJet		37.0 ^b, E, 4^	15.5	7.7	53.7	39.7 ^ab, E, 4^	8.0	29.2	56.2
SiC		40.9 ^b, F, 6^	16.0	29.3	52.7	42.3 ^a, F, 6^	16.1	30.4	61.8
HF	500 TC	33.7 ^c, A, 1^	14.2	14.8	48.6	37.0 cd^, A, 1^	13.3	13.0	47.8
MEP		17.7 ^d, B, 2^	9.6	12.4	32.1	15.2 ^c, B, 3^	22.9	4.4	43.4
CoJet		40.6 ^c, C, 4^	11.8	27.6	53.1	36.3 ^d, C, 4^	16.5	27.3	60.5
SiC		35.3 ^c, D, 6^	20.0	20.1	57.3	32.1 cd^, D, 6^	41.9	0.0	60.2
HF	90 days	32.6 ^e, A, 1^	14.8	17.3	41.9	33.7 ^e, A, 1^	14.4	19.4	59.4
MEP		33.3 ^e, B, 3^	21.7	15.8	55.9	30.2 ^e, B, 2, 3^	9.7	19.9	43.6
CoJet		31.6 ^e, C, 5^	12.2	18.1	38.5	19.0 ^e, D, 5^	16.1	8.6	35.5
SiC		35.2 ^e, E, 6^	21.5	9.6	48.2	0.40 ^f, F, 7^	4.4	0.0	5.8

Different lowercase letters indicate significant differences within the aging groups (24 h, 500 TC, 90 days) after different pretreatments in the columns, different uppercase letters indicate significant differences in the raw, and different numbers indicate significant differences within the pretreatment groups (HF, MEP, CoJet, SiC) after different aging methods in the columns. *HF* hydrofluoric acid, *MEP* Monobond Etch&Prime, *SiC* silicium carbide grinder, *TC* thermal cycling

Within LiSi ceramic, pretreatment with MEP (35.7 MPa) resulted in lower initial SBS values than pretreatment with HF (40.4 MPa, *p* = 0.032) and SiC (42.3 MPa, *p* = 0.013). After 500 thermal cycles, pretreatment with MEP (15.2 MPa) led to lower SBS values than with CoJet pretreated ones (36.3 MPa, *p* = 0.007). After 90 days of storage, pretreatment with SiC resulted in lower SBS values (0.4 MPa) compared to remaining pretreatment methods (HF: 33.7 MPa, MEP: 30.2 MPa, CoJet: 19.0 MPa; *p* < 0.001).

### Influence of artificial aging

Within the LEU/MEP group, 90 days of wet storage (33.3 MPa) resulted in higher SBS values compared to 500 thermal cycles (17.7 MPa, *p* = 0.002). For CoJet pretreatment, SBS values were lower after 90 days of storage (31.6 MPa) than the initial (37.0 MPa, *p* = 0.032) and after 500 thermal cycles (40.6 MPa, *p* = 0.008).

For LiSi combined with MEP pretreatment, 500 thermal cycles (15.2 MPa) presented lower SBS values compared to initial ones (35.7 MPa, *p* = 0.021). For CoJet and SiC pretreatment, 90 days of storage resulted in lower SBS values in comparison to the baseline (CoJet: 90 days: 19.0 MPa and initial: 39.7 MPa, *p* < 0.001; SiC: 90 days: 0.4 MPa and initial: 42.3 MPa, *p* < 0.001).

### Influence of ceramic

Within HF and MEP pretreatment, LEU showed lower initial SBS values (HF: 33.6 MPa, MEP: 23.2 MPa) than LiSi (HF: 40.4 MPa, *p* = 0.023 and MEP: 35.7 MPa, *p* = 0.003). After 90 days of wet storage and CoJet (LEU: 31.6 MPa and LiSi: 19.0 MPa, *p* = 0.004) and SiC pretreatment (LEU: 35.2 MPa and LiSi: 0.4 MPa, *p* < 0.001) presented LEU higher SBS compared to LiSi.

### Bonding to enamel

The initial SBS value was the highest (54.0 MPa) and was reduced to 39.8 MPa and 33.3 MPa after 500 thermal cycles and 90 days of storage, respectively.

### Adhesive remnant index (ARI)

On the LEU surface an ARI 3 was most frequent after pretreatment with HF, CoJet, and SiC (95.6%, 86.7%, 73.3%). ARI 0 was not detected. After pretreatment with MEP on 42.2% no adhesive, and 33.3% more than 50% remained on the surface.

After pretreatment of LiSi with HF and CoJet an ARI 3 was the most common (77.8%, 51.1%). The pretreatment with MEP resulted in an evenly distributed frequency of ARI 0, 1, and 3. In the SiC group none of the attachment material remained on the surface after SiC pretreatment (51.1%), followed by less than 50% (26.7%). On the enamel surface all the attachment material was present in 42.2% (Table 4).

**Fig. 3:**
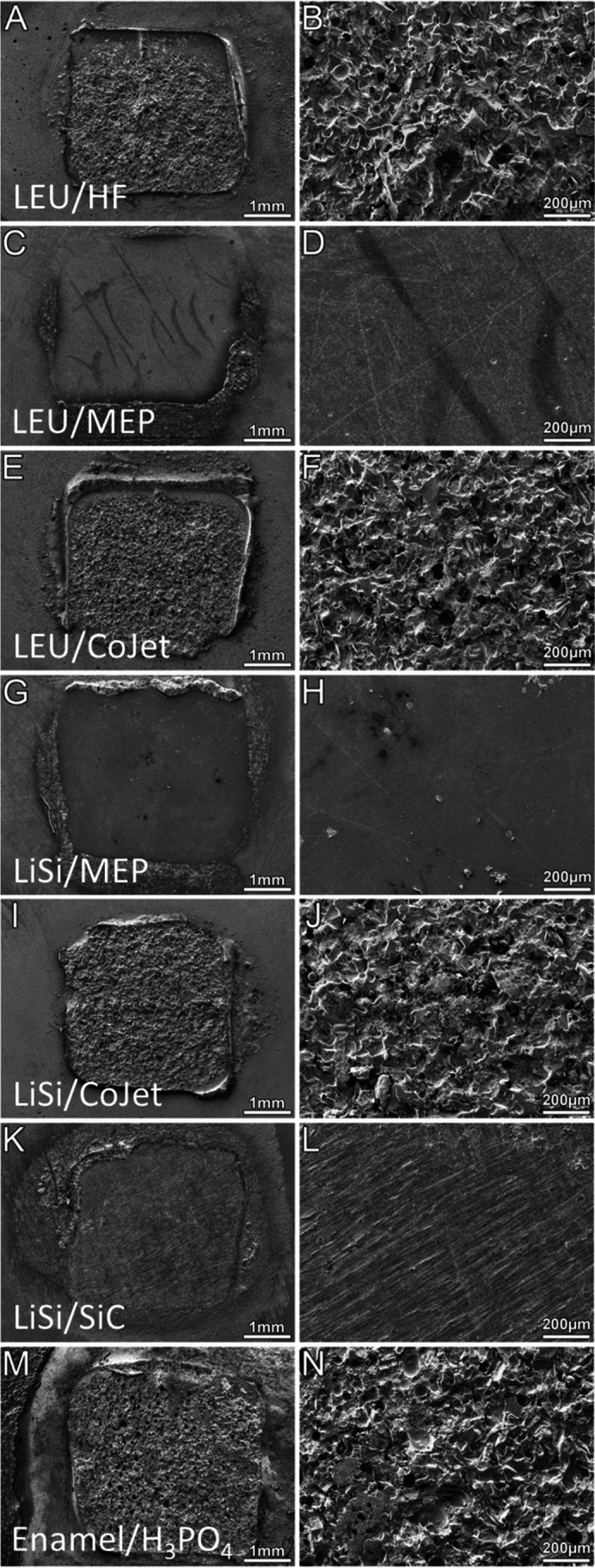
Scanning electron micrographs of the bonding areas on the ceramic surface after SBS measurement. General views at 30-fold magnification. Trapezoidal bonding areas with no attachment material left (**C**, **G**, and **K**) or all (**A**, **I**, and **M**) or almost all (**E**) of the attachment material left (left column). Close up views (250-fold) of the corresponding bonding area. A rough surface of the attachment material is discernible (**B**, **F**, **J**, and **N**) (right column). The ceramic surfaces show very little (**H**), some (**D**), and many scratches (**L**). **A** and **B** LEU/HF. **C** and **D** LEU/MEP. **E** and **F** LEU/CoJet. **G** and **H** LiSi/MEP. **I** and **J** LiSi/CoJet. **K** and **L** LiSi/SiC. **M** and **N** Enamel/phosphoric acid. *LEU* leucite, *LiSi* lithium disilicate, *HF* hydrofluoric acid, *MEP* Monobond Etch&Prime, *SiC grinder* silicium carbide grinder, *H*_*3*_*PO*_*4*_ phosphoric acid

Scanning electron microscopic images of the bonding surface on the ceramic or enamel after the SBS measurement are displayed in Fig. 3. The trapezoidal bonding area was easily discernible in all specimens.

**Table 4:** Distribution of ARI. Number and percentage of specimen and the rated ARI score (0–3). Data are given for every surface and the respective pretreatment separately and for all specimens together

Surface	Pretreatment	ARI				Total
		**0**	**1**	**2**	**3**	
LEU	HF	0 (0.0%)	1 (2.2%)	1 (2.2%)	43 (95.6%)	45 (100%)
	MEP	19 (42.2%)	15 (33.3%)	4 (8.9%)	7 (15.6%)	45 (100%)
	CoJet	0 (0.0%)	1 (2.2%)	5 (11.1%)	39 (86.7%)	45 (100%)
	SiC	0 (0.0%)	2 (4.4%)	10 (22.2%)	33 (73.3%)	45 (100%)
LiSi	HF	0 (0.0%)	2 (4.4%)	8 (17.8%)	35 (77.8%)	45 (100%)
	MEP	15 (33.3%)	14 (31.1%)	2 (4.4%)	14 (31.1%)	45 (100%)
	CoJet	3 (6.7%)	13 (28.9%)	6 (13.3%)	23 (51.1%)	45 (100%)
	SiC	23 (51.1%)	12 (26.7%)	3 (6.7%)	7 (15.6%)	45 (100%)
Enamel	H_3_PO_4_	3 (6.7%)	13 (28.9%)	10 (22.2%)	19 (42.2%)	45 (100%)
Total		63 (15.6%)	73 (18.0%)	49 (12.1%)	220 (54.3%)	405 (100%)

*HF* hydrofluoric acid, *MEP* Monobond Etch&Prime, *SiC* silicium carbide, *LiSi* lithium disilicate, *H*_*3*_*PO*_*4*_ phosphoric acid

### Reliability of measurements

The intrarater and the interrater reliability ARI assessment were both almost perfect (*κ* = 0.85) [39].

## Discussion

The aim of this in-vitro investigation was to test the SBS of ceramic brackets bonded to two different types of silicate ceramic after four different pretreatment methods and after different aging methods. The first hypothesis (“the pretreatment of the silicate ceramic shows no impact on SBS values”) had to be rejected, as pretreatment with MEP resulted in lower SBS values after 24 h and 500 thermal cycles, with a difference in the LiSi group after 24 h and in LEU group after 500 thermal cycles. After 90 days of wet storage, there were lower SBS values after pretreatment of LiSi with SiC. In the literature HF still seems to be considered as the gold standard for bonding brackets to silicate ceramic [22, 29]. However, the intraoral application of HF might be associated with some risk factors since it is known to be very toxic and hazardous. Furthermore, the different etching times for LEU and LiSi should be considered and presuppose that the clinician/patient knows the type of material used for the restoration. MEP combines the hydrofluoric-acid etching and silanization steps and contains ammonium polyfluoride for the etching effect and trimethoxypropyl methacrylate for silanization. When comparing the etching patterns between conventional etching using HF and MEP, MEP generally resulted in a less roughened surface than HF etching [40]. In prosthodontic studies, MEP showed good long-term bonding properties on different ceramic materials [31, 41]. However, very little clinical data on MEP are currently available. Two studies showed satisfactory results for this adhesive after 6 months of clinical testing [41, 42], but further studies are needed to confirm these outcomes. The authors are aware of only one further study on MEP in orthodontic research with respect to milled silicate ceramic restorations. Similar to our findings González-Serrano et al. described lower SBS values after conditioning of LiSi with MEP in comparison to HF, but the differences were not significant after 24 h. They also found a very little reduction of SBS after HF treatment and thermal cycling, but a significant reduction after MEP pretreatment [43]. In contrast to our finding, Duygu et al. reported slightly higher SBS values after pretreatment of LEU with MEP in comparison to HF [44]. Many studies comparing different pretreatment methods used airborne particle abrasion with alumina powder as an alternative for HF [45, 46]. With this kind of pretreatment there is a considerable risk to generate cracks in the ceramic. Therefore, ceramic specimens were pretreated with silica-coated aluminum oxide (CoJet) in the present study since due the coating with silica the particles are round without sharp-edged areas that could damage the ceramic [47]. Karan et al. reported similar SBS values after pretreatment of different silicate ceramics with either CoJet or 50-μm aluminia oxide sandblasting followed by HF and thermo cycling [48]. In a further investigation pretreatment of LEU with CoJet resulted in significant higher SBS values after thermal cycling compared to HF [49], and slightly higher after 24 h [44] which is in accordance with the present results, although the difference we found were not significant. All of the above described studies used metal brackets and not ceramic, as in the present investigation. Therefore, comparisons should be made with caution and especially SBS values cannot be compared. In prosthetic dentistry SiC grinders and silane (Cimara system) is one of the recommended possibilities for intraoral reparation of ceramic restorations [50]. The authors are not aware of any study investigating the use of SiC grinders (Cimara system) for orthodontic purposes and therefore the results cannot be compared. The present results indicate that SiC grinders can be recommended only for the pretreatment of LEU, as the SBS values of LiSi and the brackets were not sufficiently high after 90 days of wet storage.

The second hypothesis “the aging level shows no impact on SBS values” was rejected. While all ceramic specimens pretreated with HF and LEU specimens pretreated with SiC presented no impact of aging, pretreatment with MEP or CoJet led to a decrease or increase of SBS values. In this study, the specimens were tested initially, aged for 90 days in distilled water or aged for 500 cycles in accordance with DIN 13,990:2017–04 [33] in a thermocycling machine, which corresponds to a period of approximately 4–6 months in vivo [51, 52]. Thermocycling means a repeated cycling between two temperatures (5 and 55 °C) subject to an adequate dwelling time (20 s) to ensure thermal adjustment of the specimens without an exposure to extreme thermal stress [53]. Our measured bond strength results showed an effect only for pretreatment with MEP, regardless of the used silicate ceramic. Other studies also observed an impact of thermocycling on bond strength [54, 55]. Although in-vitro thermocycling subjects all specimens to standardized and reproducible stress, there is no systematic standard procedure for subjecting materials to cycling regimens at present. Thermal loading may lead to mechanical stress at the bonding area, causing volumetric changes. Nevertheless, thermal aging is usually still not used in numerous orthodontic bond strength tests [44, 45, 56, 57]. Ninety days of wet storage resulted in decreased SBS values between the bracket and LiSi after pretreatment with CoJet. These values were still sufficiently high for clinical use, in contrast to the SiC-pretreated LiSi. The decrease in SBS values is likely to be caused by hydrolysis, degradation of fillers, and softening [58]. The results of the present study show that in SBS testing aging in wet environment is strongly recommended to be included in addition to thermal cycling and the initial measurement. To the best of the authors’ knowledge this aspect has not been considered in any orthodontic SBS study before.

The third hypothesis “the choice of silicate ceramic shows no impact on SBS values” was rejected as well. The influence of the ceramic type was most obvious after 90 days of storage, within specimens pretreated with SiC; SBS values were below clinically acceptable limits, when brackets were bonded to LiSi. SBS values of LiSi were also decreased after CoJet application. The elastic modulus of LiSi is higher compared to LEU (approximately 95 GPa vs. 80 GPa). As a consequence, there is a higher risk for cracks during mechanical pretreatment where water is likely to be absorbed during wet storage resulting in lower SBS values. In contrast initial values were higher for LiSi after the use of HF and MEP likely due to higher surface roughness values after etching.

The results of the evaluation of the ARI are in accordance with the SBS values. An ARI of 3 (“all of the attachment material left on the ceramic”) was most common in those groups with higher SBS values (HF, CoJet, and SiC on LEU), whereas lower ARI was present in those groups with lower values such as MEP. In general, in the present study SBS values were remarkably high (median: 33.6 MPa). This might be explained by the fact that ceramic brackets were bonded to ceramic surfaces [59]. Al-Hity et al. found significant higher tensile bond strength values for ceramic brackets bonded to glass ceramic in comparison to metal brackets [2]. Conversely, this does not mean that due to these high values and the high presence of an ARI 3 there must be a high risk of damaging the ceramic surface during debonding of the brackets. Considering that in the shear bond strength testing device the debonding load is applied in only one direction (occlusal-gingival) whereas the ceramic bracket used in this investigation has an integrated stress concentrator that makes debonding by a gentle squeezing with the corresponding plier very simple. Most other ceramic brackets have special debonding mechanisms as well, and the force applied in the testing device is not comparable in any way to the in-vivo debonding mechanism. This is a considerable disadvantage of SBS testing, and evaluation of ARI and clinical consequences should be drawn very carefully. Initial SBS values between the bracket and enamel showed the highest values and decreased due to artificial aging, comparable to pretreatment with HF. Therefore, it can be assumed that bonding between ceramic surfaces and ceramic brackets can be as reliable as between human enamel and ceramic brackets.

In summary, despite some differences, almost all SBS values in the present investigation were above the required 5–10 MPa, with exception of the SiC-pretreated LiSi after 90 days of storage. Due to its in-vitro character and therefore the absence of saliva and chewing force as well as force exerted by an orthodontic wire SBS testing has several limitations. As the force is only applied from one direction the shear stress is very likely not uniform across the whole interface. Therefore, it might be advantageous reporting the debonding force under shear loading conditions. Bishara et al. reported a study setup in which the debonding force was applied via a plier from two sides of the bracket base, similar to the clinical application [60]. But most studies in the literature still use the classical SBS testing setting and also report SBS values, which is to date also recommended by the DIN 13,990:2017–04 [33]. Furthermore, results of different studies are difficult to compare due to various experimental setups. In the present study different pretreatment methods were compared to a method that has been successfully applied in clinical treatment. Therefore, the results are of clinical relevance as they can be related to a method that is already used providing a reliable basis for clinical trials, although the absolute values are not transferable to a clinical setup.

## Conclusion

Within the limitations of this in-vitro study, the following conclusions were drawn:

- The intraoral application of HF for orthodontic bonding is not necessary.
- CoJet and MEP are considerable pretreatment methods to bond ceramic brackets to silicate ceramics.
- SiC grinders should only be used for LEU pretreatment.
- Bonding on enamel presented higher or comparable values compared to bonding on ceramic restorations.
